# N_2_ activation on a molybdenum–titanium–sulfur cluster

**DOI:** 10.1038/s41467-018-05630-6

**Published:** 2018-08-10

**Authors:** Yasuhiro Ohki, Keisuke Uchida, Mizuki Tada, Roger E. Cramer, Takashi Ogura, Takehiro Ohta

**Affiliations:** 1 Department of Chemistry, Graduate School of Science, Nagoya University, Furo-cho, Chikusa-ku, Nagoya, 464-8602 Japan; 2 Research Center for Materials Science and Integrated Research Consortium on Chemical Sciences, Nagoya University, Nagoya, 464-8602 Japan; 3 Department of Chemistry, University of Hawaii, Honolulu, HI 96822 USA; 4 Picobiology Institute, Graduate School of Life Science, University of Hyogo, RSC-UH LP Center, Hyogo, 679-5148 Japan

## Abstract

The FeMo-cofactor of nitrogenase, a metal–sulfur cluster that contains eight transition metals, promotes the conversion of dinitrogen into ammonia when stored in the protein. Although various metal–sulfur clusters have been synthesized over the past decades, their use in the activation of N_2_ has remained challenging, and even the FeMo-cofactor extracted from nitrogenase is not able to reduce N_2_. Herein, we report the activation of N_2_ by a metal–sulfur cluster that contains molybdenum and titanium. An N_2_ moiety bridging two [Mo_3_S_4_Ti] cubes is converted into NH_3_ and N_2_H_4_ upon treatment with Brønsted acids in the presence of a reducing agent.

## Abstract (web summary)

Nitrogenase—whose cofactor consists of a metal–sulfur cluster—catalyzes the production of NH_3_ from N_2_, but designing metal–sulfur complexes capable of promoting this conversion remains challenging. Here, the authors report on the activation of N_2_ by a metal–sulfur cluster containing [Mo_3_S_4_Ti] cubes, demonstrating NH_3_ and N_2_H_4_ production.

## Introduction

**Fig. 1:**
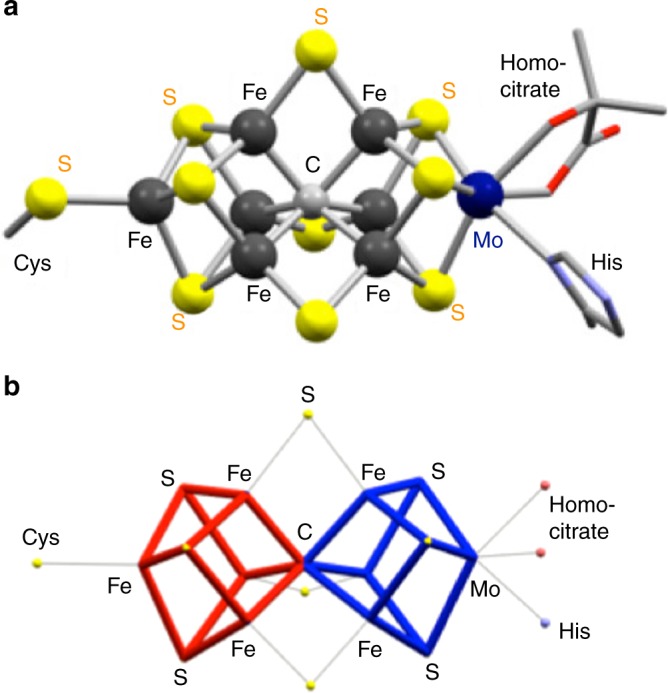
Structure of the nitrogenase FeMo-cofactor. **a** The resting-state structure. The peptide chains of cysteine (Cys) and histidine (His), as well as a part of (*R*)-homocitrate are omitted for clarity. Color legend: C, gray; Fe, black; Mo, dark blue; S, yellow; O, red; N, light blue. **b** Wire-frame drawing of the inorganic part of the FeMo-cofactor, which highlights the fused form of the two cubes

Metal–sulfur clusters are a ubiquitous class of inorganic compounds, and span a wide range of structural and electronic features^1,2^. One unique function of protein-supported clusters is the conversion of N_2_ into NH_3_, which is mediated by the FeMo-cofactor of nitrogenase (Fig. 1a)^3–5^. The FeMo-cofactor can be extracted from the protein into organic solvents without significant degradation of its metal–sulfur core^6,7^. The extracted form catalyzes the reduction of non-native C_1_ substrates, such as CO and CO_2_ in the presence of reducing agents and protons to furnish CH_4_ and short-chain hydrocarbons^8,9^, and similar catalytic reductions can also be accomplished with the synthetic analog [Cp*MoFe_5_S_9_(SH)]^3–^ (Cp* = η^5^-C_5_Me_5_)^10^. However, N_2_ cannot be reduced with the extracted FeMo-cofactor^7^ or its synthetic analogs, highlighting the difference in N_2_-reducing activity between the protein-supported FeMo-cofactor and discrete metal–sulfur clusters in solution. Thus, a molecular basis for the reduction of N_2_ on metal–sulfur clusters remains elusive, while some aqueous suspensions or emulsions of Fe–S or Mo–Fe–S compounds have been found to generate NH_3_^11–14^ and some thiolate-supported Fe complexes have been found to activate N_2_^15–17^. The cuboidal cluster Cp*_3_Ir_3_S_4_Ru(tmeda)(N_2_) (tmeda = tetramethylethylenediamine) is the only structurally characterized example for the binding of N_2_ to a metal–sulfur cluster, although the conversion of the Ru-bound N_2_ has not yet been achieved^18^.

**Fig. 2:**
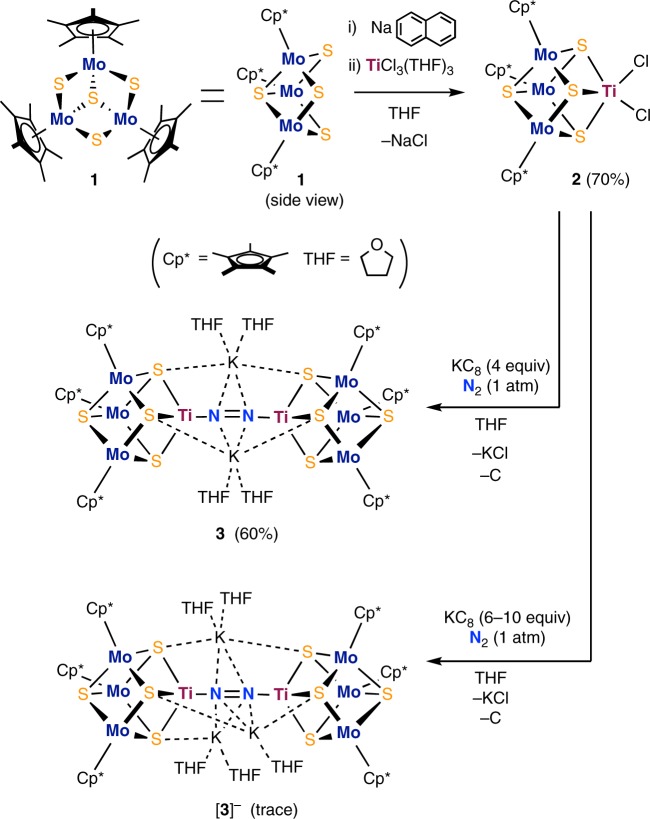
Reactions in this study. Synthesis of the cubic [Mo_3_S_4_Ti] cluster **2**, the N_2_-binding cluster **3**, and its 1e reduced form [**3**]^–^; Cp* = η^5^-pentamethylcyclopentadienyl; THF tetrahydrofuran

It should be noted that the structure of the FeMo-cofactor can be seen as a fused form of two cubes (Fig. 1b), implying the utility of cubes in the activation of N_2_. However, synthetic examples are limited for the fused form of such metal–sulfur cubes^19–23^, and a carbon-centered derivative still remains unprecedented. In order to develop a strategy for the activation of N_2_ on synthetic metal–sulfur clusters, we examined independent cubes in this study. Our design (Fig. 2) is based on the trinuclear Mo–sulfur cluster platform Cp*_3_Mo_3_(μ-S)_3_(μ_3_-S) (**1**)^24^ for the accommodation of a metal atom via three doubly bridging sulfur atoms to furnish a cubic structure. Ti was chosen as the additional metal to demonstrate the proof-of-concept, on the basis of successful examples of Ti complexes in N_2_ chemistry^25^, e.g. some recent studies on the activation of N_2_ mediated by Ti–hydride complexes^26,27^. The resulting [Mo_3_S_4_Ti] cluster (Fig. 2, top right) is reduced in the presence of N_2_, to generate a Ti-based reaction site for the activation of N_2_. Herein, we report the activation of N_2_, as well as its conversion into NH_3_ and N_2_H_4_, on a Mo–Ti–S cluster.

## Results and Discussion

### Synthesis of a cubic [Mo_3_S_4_Ti] cluster and an N_2_-cluster

**Fig. 3:**
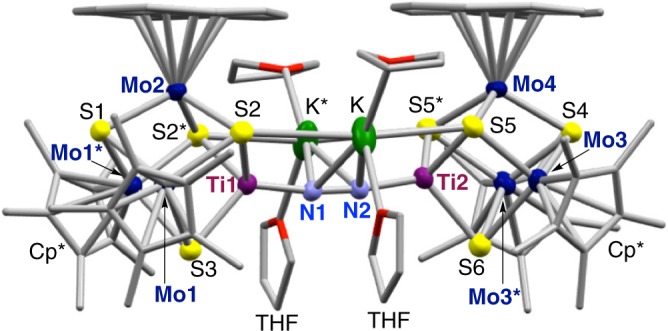
Molecular structure of the N_2_ cluster **3**. For clarity, carbon and oxygen atoms are drawn as capped sticks, while other atoms are drawn with thermal ellipsoids set at 50% probability. Color legend: C, gray; Mo, dark blue; Ti, purple; S, yellow; O, red; N, light blue; K, green. Selected bond distances (Å): N1–N2 1.294(7), Mo1–Mo1* 2.6766(8), Mo1–Mo2 2.8853(6), Mo3–Mo3* 2.7392(8), Mo3–Mo4 2.8518(6), Mo1–Ti1 3.0315(10), Mo2–Ti1 3.0908(12), Mo3–Ti2 3.0301(10), Mo4–Ti2 3.0778(12), Ti1–N1 1.802(5), Ti2–N2 1.798(5), K–N1 2.797(3), K–N2 2.782(3)

**Fig. 4:**
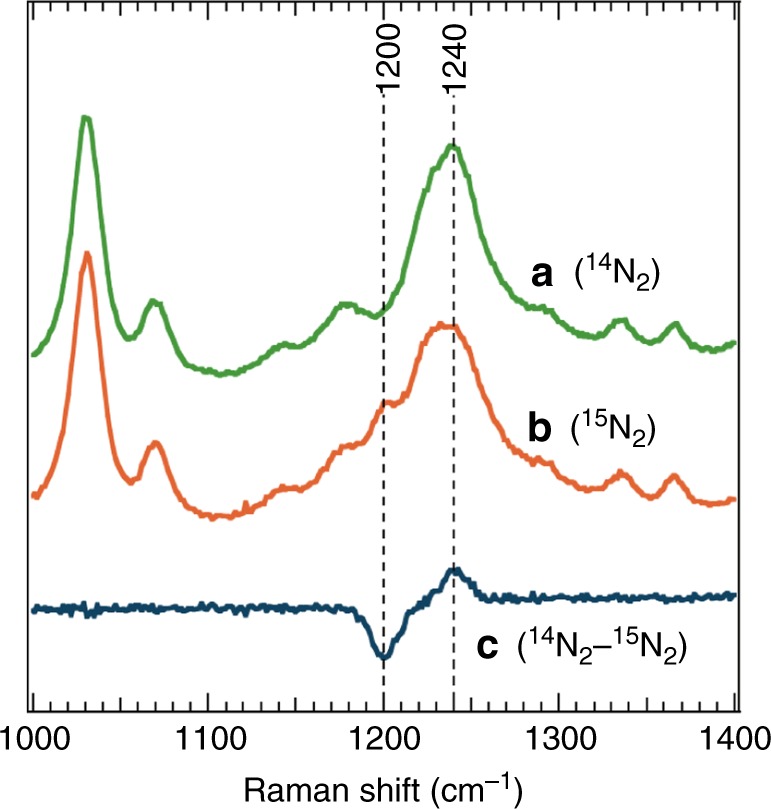
Spectroscopic characterization of the Ti-N=N-Ti moiety. Resonance-Raman spectra for **3** (**a**, green line) and ^15^N-labeled **3** (**b**, orange line), as well as their difference spectrum (**c**, dark blue line) in THF at –30 °C

The cubic [Mo_3_S_4_Ti] precursor cluster Cp*_3_Mo_3_S_4_TiCl_2_ (**2**) was synthesized in 70% yield in tetrahydrofuran (THF) from the reaction of in situ generated [**1**]^–^ with TiCl_3_(THF)_3_ (Fig. 2), in a manner similar to the synthesis of cubic clusters from trinuclear Mo–sulfur clusters^28,29^. The molecular structure of **2** (Supplementary Figure 1 and Supplementary Table 3) reveals a nearly square-pyramidal geometry of titanium with an apical sulfur atom, where the Ti–S_apical_ distance (2.2799(7) Å) is shorter than the Ti–S_basal_ distances (2.3888(6) and 2.4272(6) Å). The Ti–Cl distances (2.3429(7) and 2.3812(7) Å) are close to those in a Ti(III) complex (average 2.353(4) Å) supported by three chlorides and a tridentate phosphine ligand^30^. The reduction of **2** with potassium graphite (KC_8_) in THF under an atmosphere of N_2_ resulted in the coordination and reduction of N_2_ to form the brown cluster [K(THF)_2_]_2_[Cp*_3_Mo_3_S_4_Ti]_2_(μ-N_2_) (**3**) in 60% yield, in which an N_2_ moiety bridges two [Mo_3_S_4_Ti] cubes via binding to the Ti atoms. Product(s) of analogous reactions under atmospheres of H_2_ or Ar have not been characterized so far. Cluster **3** is diamagnetic, and the ^15^N NMR spectrum of ^15^N-labeled **3** exhibited a signal at –75.4 ppm relative to CH_3_NO_2_ in THF-*d*_8_ (Supplementary Figure 5). A single-crystal X-ray diffraction analysis of **3** established that N_2_ is linearly aligned with the two Ti atoms to form a Ti–N=N–Ti moiety (Fig. 3 and Supplementary Figure 4). Its N–N distance (1.294(7) Å) is comparable to the longest bonds reported for Ti–N=N–Ti complexes^31–33^, suggesting a high degree of N_2_ reduction. The N–N distance in **3** is slightly longer than that in H_3_CN=NCH_3_ (1.25 Å)^34^, but shorter than that in H_2_N–NH_2_ (1.46 Å)^25^, indicating a character between N=N double and N–N single bonds for the bridging N_2_ in **3**. This notion was supported by resonance-Raman spectroscopy (Fig. 4 and Supplementary Figure 8), which showed a *ν*_NN_ band of **3** at 1240 cm^−1^ based on the difference spectrum of **3** and ^15^N-labeled **3**. This *ν*_NN_ frequency falls between those of H_3_CN=NCH_3_ (1575 cm^−1^)^35^ and H_2_N–NH_2_ (1111 cm^−1^)^36^. The observed shift (40 cm^−1^) between **3** (1240 cm^−1^) and ^15^N-labeled **3** (1200 cm^−1^) is consistent with the calculated shift (42 cm^−1^) for the replacement of ^14^N_2_ by ^15^N_2_ assuming  a diatomic harmonic oscillator.

Owing to the robust Cp*–Mo and Mo–S bonds in the present system, we speculate that the three Mo atoms remain intact during the activation of N_2_, leaving Ti as the reaction site in each cube. These robust bonds would also help to avoid the degradation of the cubic structure by aggregation or fragmentation.

### Conversion of the N_2_ moiety into NH_3_ and N_2_H_4_

The high degree of reduction in **3** prompted us to attempt a stoichiometric conversion of the coordinated N_2_ into NH_3_ and N_2_H_4_. Upon treatment of **3** with protons from either H_2_O or HCl, the color changed from brown to green, and NH_3_, N_2_H_4_, and [**1**]^+^ were detected. The resulting products NH_3_ and N_2_H_4_ were quantified by indophenol and azo-dye titration methods, respectively (Supplementary Tables 1 and 2)^37,38^. As summarized in Table 1 (entries 1 and 4), small to moderate amounts of NH_3_ (0.12(3)–0.29(5) equiv) and N_2_H_4_ (0.12(10)–0.60(3) equiv) were detected by protonation of **3** with an excess H_2_O or HCl. While the length of the N–N bond in **3** indicates that a reduction has occurred, additional electrons are required for the cleavage of the N–N bond to form NH_3_. Therefore **3** was treated with KC_8_ and H_2_O or HCl. In the presence of 6 equiv of KC_8_, the average amount of NH_3_ generated by protonation with H_2_O increased from 0.12(3) to 0.47(27) equiv (entry 5), while the fluctuations observed allow only a cautious comparison. The NH_3_ yields in entries 4 and 5 (HCl) are comparable and within error. The yields of N_2_H_4_ in entries 2 (0.56(10) equiv from H_2_O) and 5 (0.19(6) equiv from HCl) remained comparable to those of reactions in the absence of KC_8_. The formation of N_2_H_4_ from the Ti–N=N–Ti moiety is reasonable, as this N–N bond exhibits an N=N double and N–N single bond character. In the presence of 0–6 equiv of KC_8_, subsequent protonation by H_2_O provided approximately three times the amount of N_2_H_4_ than the reactions with HCl. Such acid-dependent selectivity may arise from the affinities of the conjugate bases (OH^–^ or Cl^–^) toward metals, where the high oxophilicity of the Ti and K atoms can facilitate the coordination of H_2_O onto these atoms, which could lead to an efficient protonation of the neighboring Ti–N=N–Ti moiety.

**Table 1:** Protonation of **3** in the presence/absence of KC_8_^a^

Entry	Proton source	KC_8_ (equiv)	NH_3_ (equiv)	N_2_H_4_ (equiv)
1	H_2_O	0	0.12(3)	0.60(3)
2	H_2_O	6	0.47(27)	0.56(10)
3	H_2_O	100	1.20(19)	n.d.^b^
4	HCl	0	0.29(5)	0.12(10)
5	HCl	6	0.43(15)	0.19(6)
6	HCl	100	0.20(8)	n.d.^b^

^a^ Yields represent the average of three runs. Standard deviations are given in parentheses

^b^ n.d. = not detected

Even in the presence of a slight excess (6 equiv) of KC_8_, conversion of the N_2_ moiety of **3** into NH_3_ was not complete and notable amounts of N_2_H_4_ were detected (entries 2 and 5). These results indicate that some of the added KC_8_ remains unreacted with the N_2_ cluster and is subsequently quenched directly by H_2_O or HCl. However, the formation of N_2_H_4_ was suppressed when a large excess of KC_8_ was added prior to protonation (entries 3 and 6). While the protonation with HCl did not increase the amount of NH_3_ (entry 6), the maximum yield of NH_3_ (1.20(19) equiv) was achieved when 100 equiv KC_8_ were added prior to the protonation with H_2_O (entry 3). When conditions identical to those of entry 3 were applied for the ^15^N-enriched **3** under an atmosphere of ^14^N_2_, the ^1^H NMR spectrum of the resulting ammonium salt indicated the exclusive formation of ^15^NH_3_, which originates from the Ti–^15^N=^15^N–Ti moiety (Supplementary Figure 15). After addition of an excess of protons to **3**, the Ti atom probably dissociates from the [Mo_3_S_4_Ti] cube, which is supported by the appearance of an intense signal of [**1**]^+^ in the electro-spray ionization mass (ESI-MS) spectrum of the resulting mixture (Supplementary Figure 16) and the color change of the mixture from dark brown to green. Thus, at this stage, the possibility of N_2_ functionalization after dissociation of the Ti atom cannot be ruled out unequivocally.

It has been reported that metal–sulfur clusters exhibit stability across various oxidation states, which allows the storage and transportation of electrons^39,40^. It is therefore worthy of notice that the 1e-reduced cluster [**3**]^–^ was obtained in trace amounts from the reaction of **2** with 6–10 equiv of KC_8_ under an atmosphere of N_2_ (Fig. 2, bottom). The characterization of [**3**]^–^ was carried out only by ^1^H NMR and crystallographic analysis due to the trace amounts available. Cluster [**3**]^–^ is paramagnetic and its ^1^H NMR spectrum in THF-*d*_8_ exhibited a broad Cp* signal at 5.84 ppm (Supplementary Figure 10). The molecular structure of [**3**]^–^ (Supplementary Figure 9) revealed an interaction of three potassium ions with the bridging N_2_ moiety, where the N–N distance (1.293(5) Å) is comparable to that in **3** (1.294(7) Å). A structural comparison between the [Mo_3_S_4_Ti] cubes of **3** and [**3**]^–^ reveals the elongation of distances in [**3**]^–^. In detail, a relatively large contribution of the metal atoms for the storage of the additional electron is indicated by the larger differences in the average distances of Mo–Ti (3.0487(12) Å for **3** and 3.0242(8) Å for [**3**]^–^) and Mo–Mo (2.8150(8) Å for **3** and 2.8668(6) Å for [**3**]^–^), rather than the average metal–sulfur distances, i.e. Mo–S (2.3952(16) Å for **3** and 2.3966(11) Å for [**3**]^–^), and Ti–S (2.328(2) Å for **3** and 2.3297(13) Å for [**3**]^–^). The detailed reaction pathways for the formation of NH_3_ and N_2_H_4_ from **3** or [**3**]^–^ remain unclear at this point, as the dissociation of the Ti atom from the [Mo_3_S_4_Ti] cube renders the identification of possible cluster intermediates difficult.

In summary, we successfully achieved the activation of N_2_ and its conversion into sub-stoichiometric mixtures of NH_3_ and N_2_H_4_ on synthetic Mo–Ti–S cubes by selectively generating the Ti-based reaction sites. This work thus represents a step towards the assembly of structural and functional models for the FeMo-cofactor.

## Methods

### General procedure

All reactions and manipulations were carried out under an atmosphere of nitrogen using standard Schlenk line or glove box techniques. Solvents for reactions were purified by passing over columns of activated alumina and a supported copper catalyst. TiCl_3_(THF)_3_^41^, [Cp*_3_Mo_3_S_4_][PF_6_] ([**1**]^+^)^42^, and KC_8_^43^ were prepared according to literature procedures. All other reagents were purchased from common commercial sources and used without further purification. General considerations on the measurements and experiments as well as some experimental details are described in the Supplementary Methods.

### Modified synthesis of Cp*_3_Mo_3_S_4_ (1)

Compound **1** was originally synthesized from the reaction of Cp*Mo(S^t^Bu)_3_ (^t^Bu = *tert*-butyl) with Na/Hg^24^, but this reaction is not ideally suitable for a gram-scale synthesis. Instead, cluster **1** was synthesized on a multi-gram scale through the reduction of [**1**]^+^. For that purpose, KC_8_ (1.36 g, 10.06 mmol) was added in portions to a THF (150 mL) suspension of [**1**]^+^ (9.78 g, 10.12 mmol) at room temperature. After stirring this mixture overnight, the solvent was removed under reduced pressure. The residue was extracted four times with toluene (350 mL). The extract was stored at –30 °C to precipitate green blocks of **1**. The mother liquor was concentrated to ca. 1/5 of the original volume and stored at –30 °C to obtain a second crop. The total amount of green crystals of **1** was 6.17 g (7.51 mmol, 83%). This product was characterized by comparison with ^1^H NMR data in the literature^24^. ^1^H NMR (C_6_D_6_): *δ* 8.56 (*w*_1/2_ = 14 Hz, Cp*).

### Synthesis of Cp*_3_Mo_3_S_4_TiCl_2_ (2)

A THF (20 mL) solution of **1** (1.00 g, 1.22 mmol) was cooled to –100 °C, and a THF solution of sodium naphthalenide (19.5 mM, 62 mL, 1.21 mmol) was added. The reaction mixture was gradually warmed to room temperature to give a dark green solution. The mixture was again cooled to –100 °C, and TiCl_3_(THF)_3_ (451 mg, 1.22 mmol) was added. The reaction mixture was warmed to room temperature and stirred for 5 days, affording a dark brown suspension. After concentration to ca. 5 mL under reduced pressure, the mixture was cooled to –30 °C, which caused the precipitation of a dark brown powder. The supernatant was removed and the dark brown powder was washed with hexane (20 mL × 2). The brown powder was dissolved into CH_2_Cl_2_ (15 + 3 mL), and hexane (100 mL) was layered on top of this solution. The subsequent slow diffusion led to the formation of brown plates of Cp*_3_Mo_3_S_4_TiCl_2_**·**CH_2_Cl_2_ (**2·**CH_2_Cl_2_). These  brown crystals were washed with hexane (15 mL × 2), and then dissolved in THF (15 mL). After stirring overnight, THF and CH_2_Cl_2_ were removed under reduced pressure to give a brown powder of **2** (801 mg, 0.854 mmol, 70%). ^1^H NMR (CD_2_Cl_2_): *δ* 9.23 (*w*_1/2_ = 8 Hz, Cp*); solution magnetic moment (CD_2_Cl_2_, 297 K): *μ*_eff_ = 1.3 μ_B_; Cyclic voltammogram (2 mM in CH_2_Cl_2_, potential vs. (C_5_H_5_)_2_Fe/[(C_5_H_5_)_2_Fe]^+^): *E*_1/2_ = –0.28, –1.34 V; UV/Vis (CH_2_Cl_2_): *λ*_max_ 415 nm (sh, 1200 cm^−1^ M^−1^), 753 nm (660 cm^−1^ M^−1^); analysis (calcd., found for C_30_H_45_Cl_2_Mo_3_S_4_Ti): C (38.31, 38.77), H (4.82, 4.70), S (13.64, 13.24).

### Synthesis of [K(THF)_2_]_2_[Cp*_3_Mo_3_S_4_Ti]_2_(μ-N_2_) (3)

Cluster **2** (200 mg, 0.213 mmol) and KC_8_ (116 mg, 0.858 mmol) were charged into one side of an H-shaped glass vessel (Supplementary Fig. 3). The other arm of the vessel was charged with THF (10 mL), which was vacuum-transferred to the solid mixture at 78 K. The vessel was filled with N_2_ (1 atm) and then sealed. The mixture was slowly warmed using a methanol/liquid N_2_ slurry. Stirring of the mixture with a magnetic stirring bar was initiated, once part of frozen THF had melted. The mixture was gradually warmed to room temperature, giving a dark red-brown suspension. Stirring was continued overnight, before the solvent was removed under reduced pressure and the residue was extracted with THF (50 mL). The solution was filtered, concentrated to ca. 3 mL, and stored at –30 °C. Cluster **3** (136 mg, 0.064 mmol, 60%) was obtained as dark-brown crystalline blocks, which were washed with pentane. ^1^H NMR (THF-*d*_8_): *δ* 1.70 (Cp*); ^13^C{^1^H} NMR (THF-*d*_8_): *δ* 13.6 (C_5_*Me*_5_), 96.3 (*C*_5_Me_5_); UV/Vis (THF): *λ*_max_ 356 nm (35800 cm^−1^ M^−1^), 500 nm (15,800 cm^−1^ M^−1^); Resonance-Raman spectrum (0.07 mM in THF, *λ*_ex_ 355 nm, –30 °C): 1240 cm^-1^ (*ν*_NN_); analysis (calcd., found for C_76_H_122_K_2_Mo_6_N_2_O_4_S_8_Ti_2_): C (42.78, 42.36), H (5.76, 5.80), N (1.31, 1.37), S (12.02, 12.27).

### Synthesis of ^15^N_2_-labeled 3

This cluster was synthesized in a manner similar to that of **3**, using cluster **2** (173 mg, 0.184 mmol), KC_8_ (100 mg, 0.740 mmol), and ^15^N_2_ (1 atm). Crystals of ^15^N_2_-labeled **3** were obtained in 60% yield (117 mg, 0.055 mmol). ^15^N{^1^H} NMR (THF-*d*_8_, referenced to external CH_3_NO_2_): *δ* –75.4; Resonance-Raman spectrum (THF, –30 °C): 1200 cm^−1^ (*ν*_NN_).

### Data availability

The X-ray crystallographic coordinates for structures reported in this study have been deposited at the Cambridge Crystallographic Data Centre (CCDC), under deposition numbers 1577330–1577332. These data can be obtained free of charge from The Cambridge Crystallographic Data Centre via www.ccdc.cam.ac.uk/data_request/cif. All other data are available from the authors upon request.

## Electronic supplementary material

Supplementary Information
