# Supplementary material for: N2 activation on a molybdenum–titanium–sulfur cluster
Source: Nat Commun. 2018 Aug 10;9:3200. doi: 10.1038/s41467-018-05630-6 (PMC6086867; doi:10.1038/s41467-018-05630-6)
Supplement: Supplementary file 1 — Supplementary Information [file 41467_2018_5630_MOESM1_ESM.pdf]

N_2 activation on a molybdenum-titanium-sulfur cluster

Ohki *et al.*

Supplementary Methods

General Considerations. All reactions were carried out under an atmosphere of nitrogen, using standard Schlenk line or glove box techniques. Toluene, hexane, pentane, tetrahydrofuran (THF), CH_2Cl_2, and diethylether (Et_2O) were purified by passing over columns of activated alumina and a supported copper catalyst supplied by Hansen & Co. Deuterated solvents were purified via repeated freeze-pump-thaw cycles, drying over appropriate reagents, and vacuum-transfer under reduced pressure. ^{1}H NMR, $^{13}C\{^{1}H\}$ NMR, and $^{15}N\{^{1}H\}$ NMR spectra were recorded on a JEOL ECA600 spectrometer. The ^{1}H and the $^{13}C\{^{1}H\}$ NMR chemical shifts are given in ppm relative to the residual signals of the deuterated solvents. The ^{15}N NMR chemical shift in THF-d_8 is referenced relative to the external standard CH_3NO_2 ($\delta = 0$). Low-temperature experiments were carried out in a glove box using a Techno Sigma UCR-150-GB. UV/Vis and UV/Vis/near IR spectra were measured on a JASCO V560 or V770 spectrometer. IR spectra were measured on a Shimadzu IR-Affinity1 spectrometer using KBr pellets or an ATR-equipped Bruker Alpha FT-IR spectrometer. Resonance Raman spectra were obtained using a liquid nitrogen cooled CCD detector (CCD-1024×256-OPEN-1LS, HORIBA Jobin Yvon) attached to a 1 m single polychromator (MC-100DG, Ritsu Oyo Kogaku) with a 1200 groovs/mm holographic grating. An excitation wavelength of 355 nm was provided by an Nd-YAG laser (Photonic Solutions, SNV-20F) with incident power of 4.7 mW at the sample point using an ~135° back scattering configuration. All measurements were carried out at –30 °C. Raman shifts were calibrated with indene, and the accuracy of the peak positions of the Raman bands was ±1 cm^{-1}. Cyclic voltammograms (CV) were

recorded in THF or CH_2Cl_2 at room temperature using glassy carbon as the working electrode with 0.1-0.2 M [^{n}Bu$_4$N][PF$_6$] as the supporting electrolyte. All potentials are referenced relative to $(C_5H_5)_2Fe/[(C_5H_5)_2Fe]^+$ (Fc/Fc$^+$). Elemental analyses were recorded on an Elementar vario MICRO cube instrument where crystalline samples were sealed in tin capsules under nitrogen. The ESR spectra were recorded on a JEOL JES-FA200 spectrometer. Labeled $^{15}N_2$ was purchased from Cambridge Isotope Laboratories. Unless otherwise noted, all other compounds were purchased from common commercial sources and used without further purification. The synthesis of clusters **2** and **3** is described in *Methods* section of the main manuscript.

Large-scale synthesis of [Cp*$_3$Mo$_3$S$_4$][PF$_6$] [1]$^+$. Under an N_2 atmosphere, Cp*Mo(S^{t}Bu)$_3$ (30.0 g, 60.2 mmol) was dissolved in CH_2Cl_2 (500 mL) and treated with ferrocenium hexafluorophospate [$(C_5H_5)_2$Fe][PF$_6$] (6.64 g, 20.1 mmol) at room temperature. The resulting brown to green mixture was stirred overnight, and the volatile materials were removed under reduced pressure. The product [Cp*$_3$Mo$_3$S$_4$][PF$_6$] was originally isolated in 74% yield by column chromatography (Takei, I. *et al. Organometallics* **22**, 1790 (2003)), but this procedure is not suitable for a multi-gram scale synthesis. Instead, the product was isolated in a slightly lower yield as a green powder, by filtration of a THF suspension of the crude material to collect the green solid, followed by repeated washing with THF (> 10 times) until the filtrate is not brown anymore (9.88 g, 10.2 mmol, 51% yield). It should be noted that the product is air-stable and sparingly soluble in THF. Therefore the collection and washing processes were carried out in air. *Caution*: All procedures need to be conducted in a fume hood as all volatile materials as well as the washings smell unpleasant. ^{1}H NMR (CDCl$_3$): δ

2.01 (Cp*).

The precursor Cp*Mo(S^{t}Bu)$_3$ was synthesized by using the previously reported procedure (Kawaguchi, H. *et al. J. Am. Chem. Soc.* **119**, 10346 (1997)), while on a large scale, *i.e.* treatment of an ice-cooled THF suspension of Cp*MoCl$_4$ (10 grams or more) with 4 equiv of LiS^{t}Bu, which was generated *in-situ* from a THF solution of HS^{t}Bu and a hexane solution of ^{n}BuLi, followed by stirring at room temperature for 1 h, evaporation of all volatile materials, extraction of the product with hexane, filtration, and concentration of the extract under reduced pressure, and followed by crystallization at –30 °C.

Formation of a trace amount of [K$_3$(THF)$_5$][Cp*$_3$Mo$_3$S$_4$Ti]$_2$(μ-N$_2$) [3]$^-$. A THF (2 mL) suspension of KC$_8$ (56 mg, 0.414 mmol) was added to a THF (4 mL) suspension of **2** (200 mg, 0.213 mmol) at room temperature, resulting in the formation of dark-brown and black precipitates as well as a light green-brown supernatant. Further addition of a THF (6 mL) suspension of KC$_8$ (116 mg, 0.821 mmol) caused a color change, furnishing an intense red-brown solution. After being stirring for 5 minutes at room temperature, the mixture was filtered, and pentane (10 mL) was added to the filtrate. Leaving the solution to stand at –30 °C led to the formation of a trace amount of **4**, which was obtained in the form of black crystalline blocks. Cluster **4** was characterized by means of X-ray crystallographic analysis. The corresponding ^{1}H NMR (Supplementary Fig. 10) was measured using a crystalline solid sample containing a small amount of impurity. ^{1}H NMR (THF-d$_8$): δ 5.84 ($w_{1/2}$ = 60 Hz, Cp*).

Protonation of cluster 3 with H$_2$O or HCl and quantification of the generated ammonia and

hydrazine (Table 1). THF (3 mL) was added to a mixture of cluster **3** (5 mg, 2.3 μmol) and varying amounts of KC_8 (0-100 equivalents relative to **3**), which were kept in one arm of an H-shaped glass vessel (hereafter referred as the '*reaction tube*') (Supplementary Fig. 3). The mixture was frozen using liquid N_2, and a 1 M THF solution of H_2O (1.0 mL, 1.0 mmol) or a 2 M Et_2O solution of HCl (0.8 mL, 1.6 mmol) was added and the reaction vessel was sealed. The mixture was gradually warmed to room temperature. After stirring the mixture overnight, all volatiles were vacuum-transferred to the other arm of the H-shaped glass vessel (hereafter referred as the '*collection tube*'). The residual solid in the *reaction tube* was treated with a THF solution of potassium *tert*-butoxide (0.8 M, 4.0 mL, 3.2 mmol), and the mixture was stirred for 5 minutes to convert all NH_4 salts into NH_3. Subsequently, the volatiles in the *reaction tube* were vacuum-transferred to the *collection tube*. An Et_2O solution of HCl (2 M, 4.0 mL, 8 mmol) was added to the *collection tube*, and the vessel was sealed and warmed to room temperature. Solvents were removed under reduced pressure to give a white solid containing ammonium and hydrazinium salts. The solid in the *collection tube* was dissolved into H_2O (10 mL) in a volumetric flask. An aliquot (2.5 mL) of this solution was subsequently analyzed colorimetrically. In order to maintain the accuracy of the analyses, calibration curves using fresh reagent solutions were recorded periodically (Supplementary Fig. 13-14); For NH_3: phenol, a sodium nitroprusside solution (**A**) and a sodium hydroxide/sodium hypochlorite solution (**B**). For N_2H_4: *para*-dimethylamino-benzaldehyde/*conc*. HCl in ethanol (**C**) and an aqueous HCl solution (**D**).

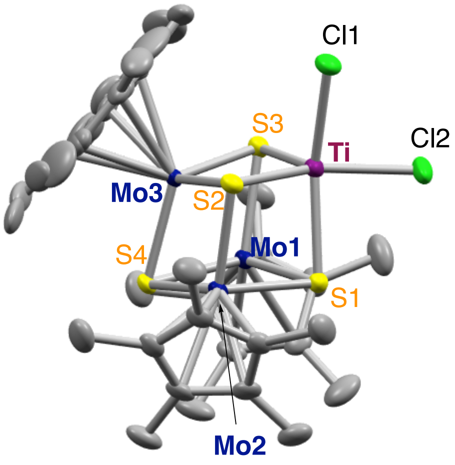

Supplementary Figure 1 Molecular structure of cluster **2** with thermal ellipsoids set at the 50% probability level. Selected bond lengths (Å): Ti-Mo1 3.0680(5), Mo1-Mo2 2.8902(4), Mo1-Mo3 2.6783(3), Mo2-Mo3 2.8630(3), Ti-Cl1 2.3812(7), Ti-Cl2 2.3429(7), Ti-S1 2.4272(6), Ti-S2 2.3888(6), Ti-S3 2.2799(7).

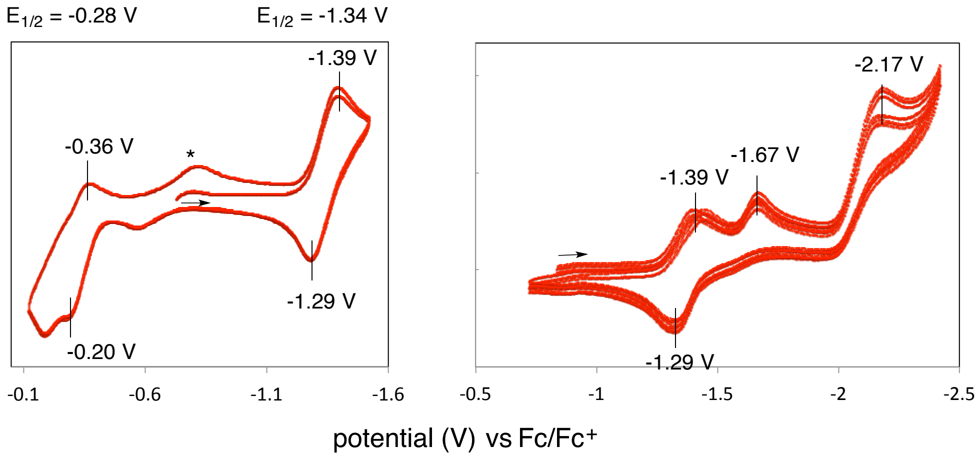

Supplementary Figure 2 Cyclic voltammogram of cluster **2** (2.0 mM) in CH_2Cl_2. Supporting electrolyte: 0.1 M [$^nBu_4N$][PF_6]; working electrode: glassy carbon; scan rate: 100 mV/s; a weak feature (*) at –0.8 V was observed only after scanning at $E_{1/2}$ = –0.28 V.

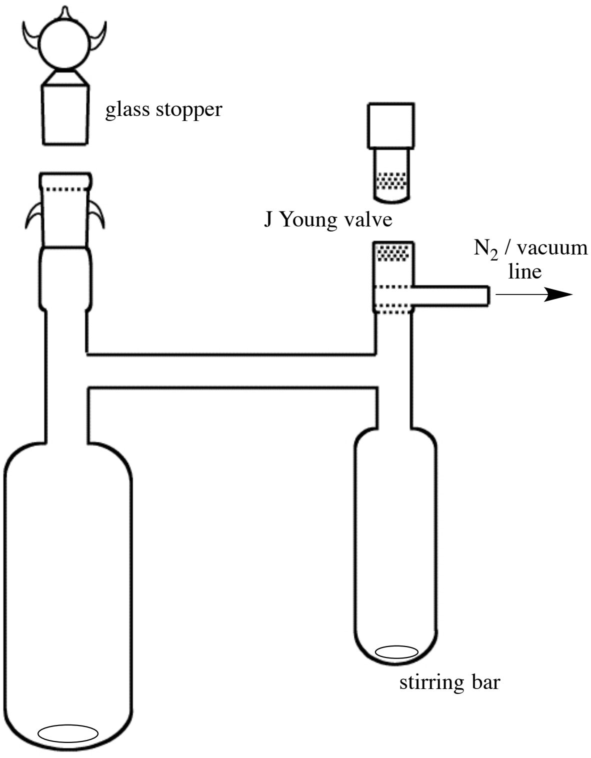

Supplementary Figure 3 The H-shaped glassware used in this study. The right arm of glass vessel is referred as the '*reaction tube*', while the left arm is referred as the '*collection tube*'.

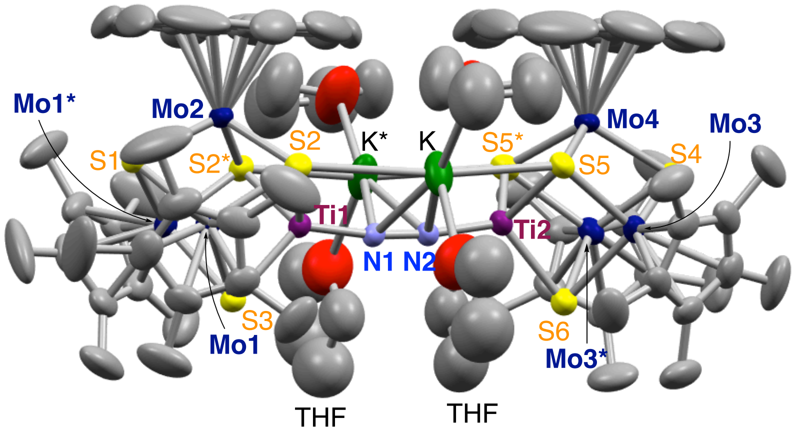

Supplementary Figure 4. Molecular structure of cluster **3** with thermal ellipsoids set at the 50% probability. Selected bond lengths (Å) and angles (°): N1-N2 1.294(7), Mo1-Mo1* 2.6766(8), Mo1-Mo2 2.8853(6), Mo3-Mo3* 2.7392(8), Mo3-Mo4 2.8518(6), Mo1-Ti1 3.0315(10), Mo2-Ti1 3.0908(12), Mo3-Ti2 3.0301(10), Mo4-Ti2 3.0778(12), Ti1-N1 1.802(5), Ti2-N2 1.798(5), K-N1 2.797(3), K-N2 2.782(3), Ti1-N1-N2 172.2(4), Ti2-N2-N1 173.1(4).

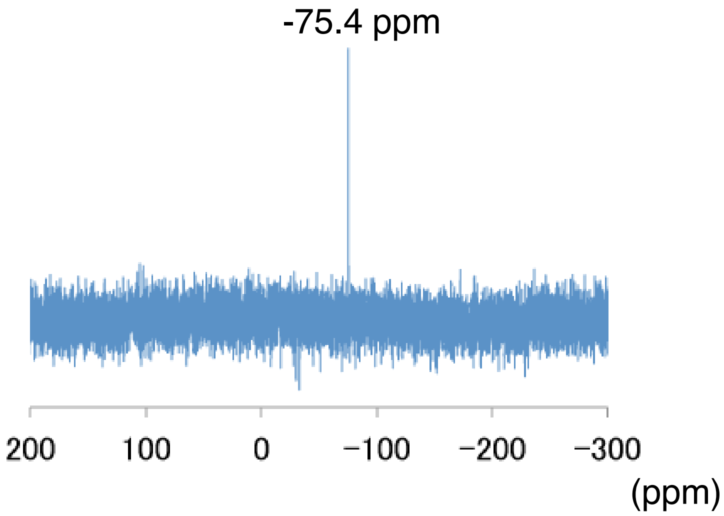

Supplementary Figure 5 $^{15}N\{^1H\}$ NMR spectrum of $^{15}N_2$-labeled **3** in THF-d_8. The chemical shift is referenced to the external standard CH_3NO_2 ($\delta = 0$).

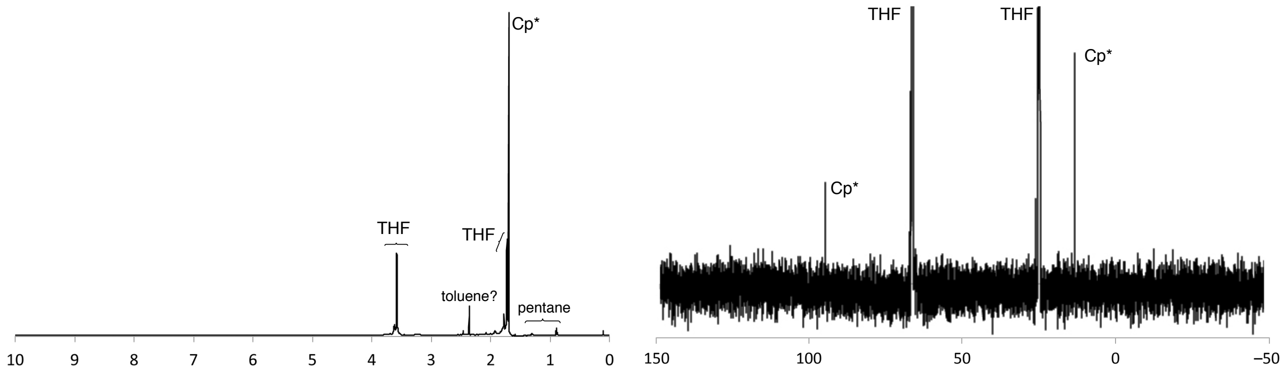

Supplementary Figure 6 ^{1}H (left) and $^{13}C\{^{1}H\}$ (right) NMR spectra of **3** in THF-d_8. The chemical shifts are given in ppm relative to the residual signals of the deuterated solvents.

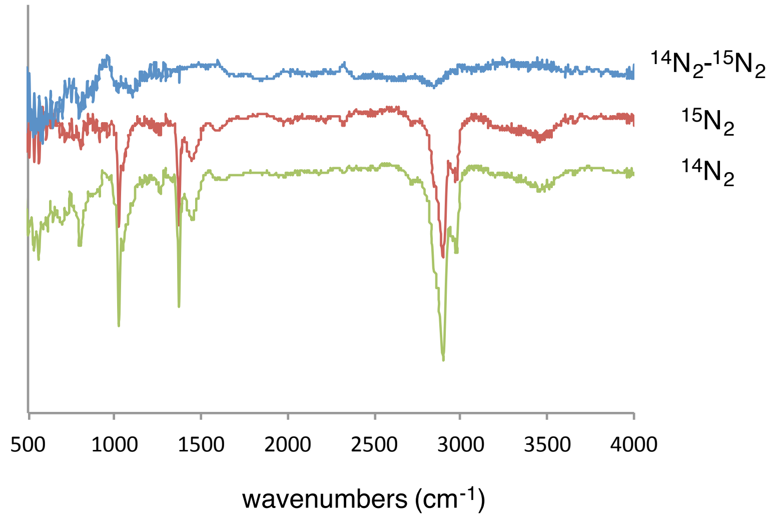

Supplementary Figure 7 Infrared (IR) spectra of **3** and $^{15}N_2$-labeled **3**, as well as their difference spectrum. It should be noted that the N-N band for the symmetric Ti-N=N-Ti moiety is inactive in the IR spectrum, and thus does not clearly appear in the difference spectrum (blue).

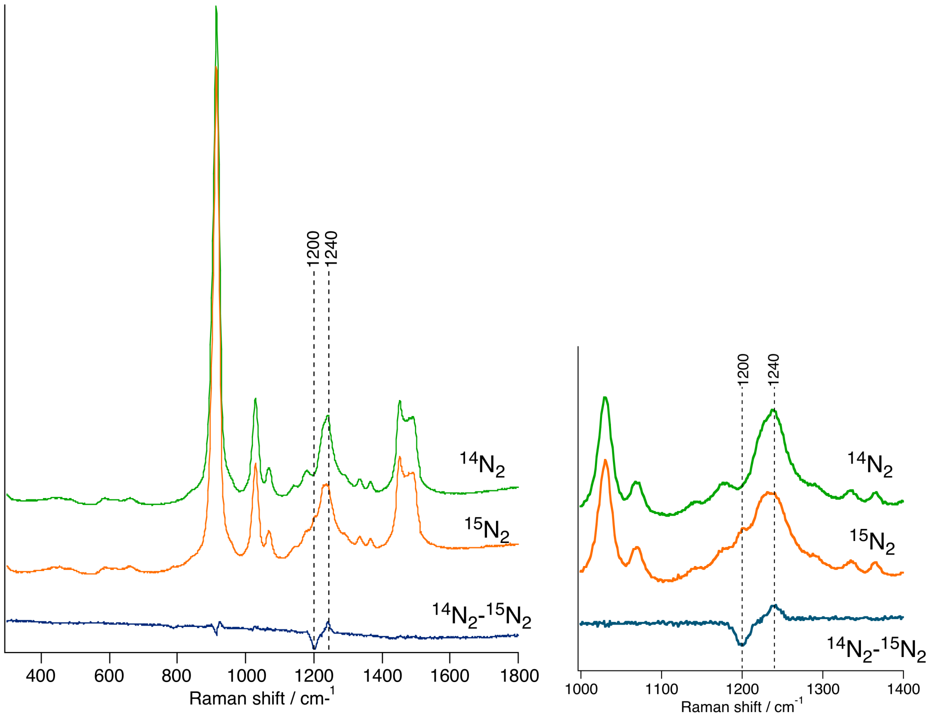

Supplementary Figure 8 Resonance-Raman spectra of **3** and $^{15}N_2$-labeled **3**, as well as their difference spectrum in THF at –30 °C: measurement conditions; λ_{ex} 355 nm, 4.7 mW, 5-10 min accumulation.

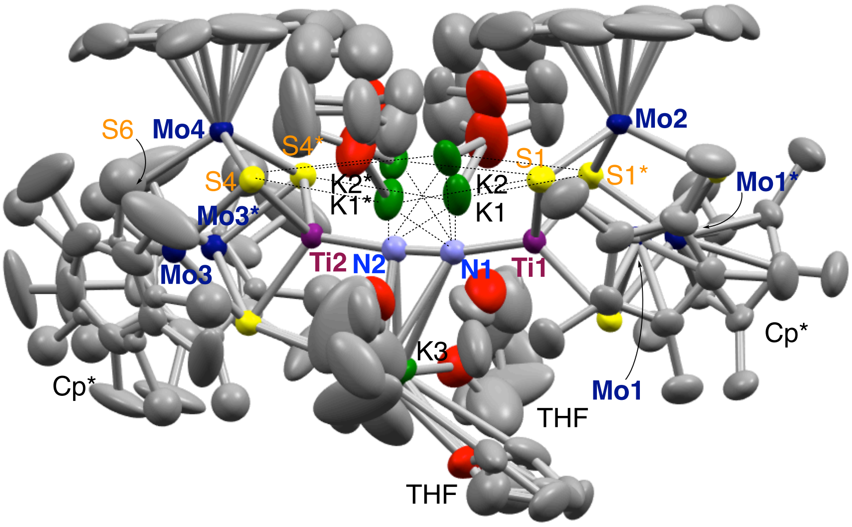

Supplementary Figure 9 Molecular structure of $[K_3(THF)_5][Cp^*{}_3Mo_3S_4Ti]_2(\mu\text{-}N_2)$ [**3**]$^-$ with thermal ellipsoids set at 50% probability. Selected bond lengths (Å) and angles (°): N1-N2 1.293(5), Mo1-Mo1* 2.7001(5), Mo1-Mo2 2.8893(4), Mo3-Mo3* 2.8455(6), Mo3-Mo4 2.9380(4), Mo1-Ti1 3.0325(7), Mo2-Ti1 3.1179(8), Mo3-Ti2 2.9890(8), Mo4-Ti2 2.9845(8), Ti1-N1 1.814(4), Ti2-N2 1.835(4), K1-S1 3.255(2), K1-S4 3.2895(19), K2-S1 3.2315(19), K2-S4 3.1834(18), K3-S5 3.348(2), K1-N1 2.774(2), K1-N2 2.742(2), K2-N1 2.818(3), K2-N2 2.747(3), K3-N1 3.014(4), K3-N2 2.715(4), Ti1-N1-N2 169.7(3), Ti2-N2-N1 173.8(3).

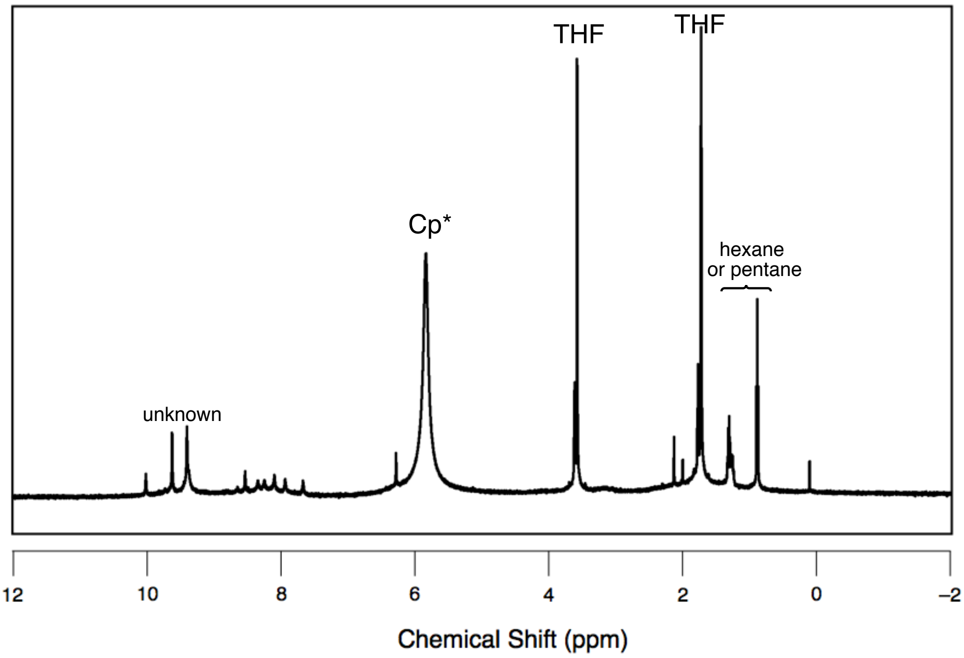

Supplementary Figure 10 ^{1}H NMR spectrum of a crystalline sample of [**3**]$^{-}$ in THF-d_8. The chemical shifts are given in ppm relative to the residual signals of the deuterated solvents.

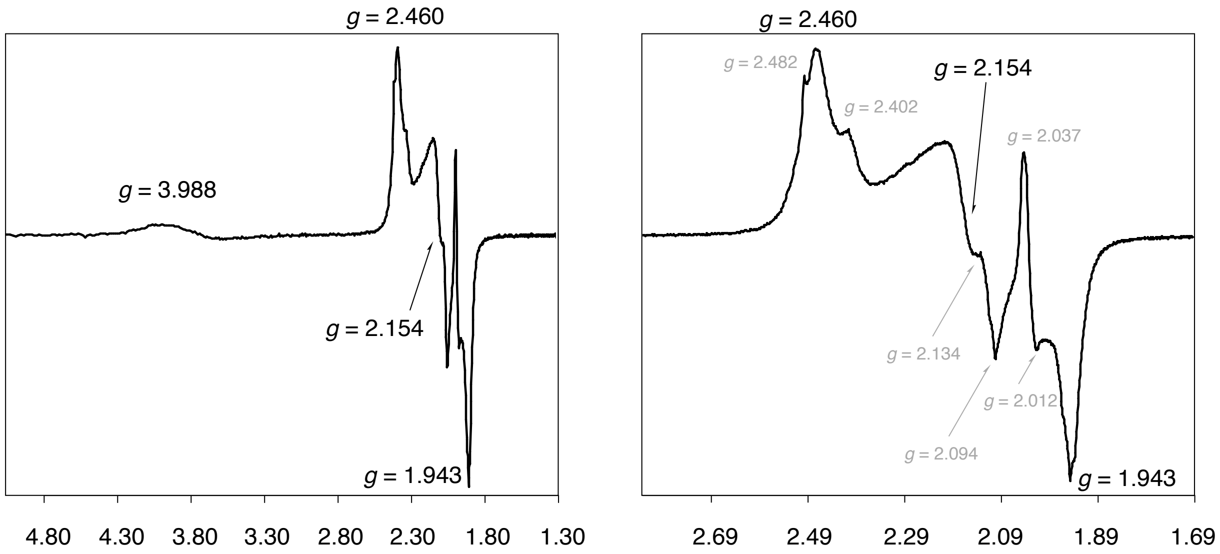

Supplementary Figure 11 Preliminary X-band ESR spectrum of cluster $[\mathbf{3}]^-$ in frozen THF at 9 K. Note that some unknown impurities exist in the sample. The major component of the ESR spectrum was tentatively assigned to a rhombic signal at g = 2.460, 2.154, 1.943, based on the similarities of the spectral shape and the g-values to those reported for the S = 1/2 signals of [8Fe-7S] clusters featuring a fused form of two cubes (*e.g.*, g = 2.209, 2.074, 1.952 for $[Fe_4S_3(SDmp)]_2(\mu\text{-}SDmp)_2(\mu\text{-}SMes)(\mu_6\text{-}S)$ (Mes = mesityl, Dmp = 2,6-(mesityl)$_2C_6H_3$); Hashimoto, T., Ohki, Y. & Tatsumi, K. *Inorg. Chem.* **49**, 6102-6109 (2010)). The presence of a higher spin state species is indicated by a feature at g = 3.988. A detailed assignment and a spectral simulation were rendered difficult by the overlapping of multiple components. Hyperfine couplings of the tentative major component with the ^{14}N (I = 1), ^{95}Mo (I = 5/2), and ^{97}Mo (I = 5/2) nuclei were not conclusive.

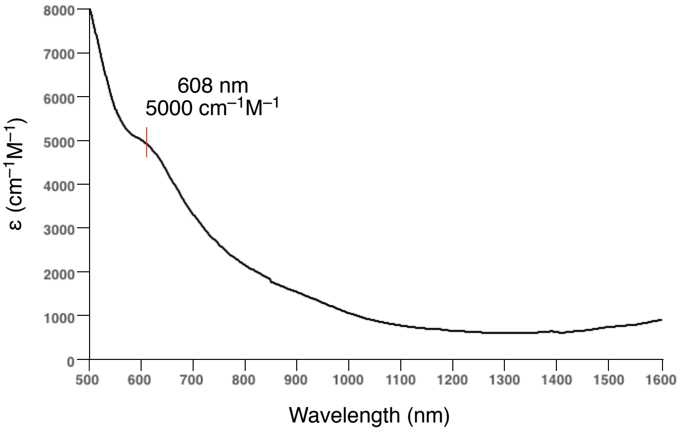

Supplementary Figure 12 Absorption spectrum of a THF solution of [**3**]$^-$ in the near-IR region. Cluster [**3**]$^-$ can be considered as a mixed-valence complex with a bridging N_2 ligand. However, the low-energy band typical for inter-valence transition does not appear in the near-IR region.

Supplementary Table 1 Absorption data for the quantification of NH_3 and N_2H_4 (1): Cluster **3** + H_2O. Calibration curves are shown in Supplementary Figures 13-14. For runs in the presence of 0 or 6 equiv of KC_8, the aliquots for the N_2H_4 analyses were diluted five times to bring their absorptions within the ranges of the calibration curves.

	KC8	run	abs.		NH_3 (mM)		NH_3 (μmol)		equiv. per Ti-N=N-Ti		Calibration curve	
			NH_3	N_2H_4	NH_3	N_2H_4	NH_3	N_2H_4	NH_3	N_2H_4	NH_3	N_2H_4
[Ti-NN-Ti] 5 mg (2.3 μmol) +H_2O	0 eq.	1	0.10195	0.35735	0.0357	0.0261	0.357	1.305	0.155	0.567	(a-1)	(b-1)
		2	0.10402	0.38001	0.02618	0.02771	0.2618	1.3855	0.114	0.602	(a-2)	(b-2)
		3	0.09276	0.39033	0.02335	0.02846	0.2335	1.423	0.102	0.619	(a-2)	(b-2)
	2 mg (14.8 μmol)	1	0.27452	0.37479	0.1037	0.0271	1.037	1.355	0.318	0.589	(a-3)	(b-3)
		2	0.51309	0.28475	0.1799	0.0208	1.799	1.040	0.782	0.452	(a-1)	(b-1)
		3	0.28285	0.41045	0.06775	0.02946	0.6775	1.473	0.295	0.640	(a-4)	(b-4)
	32 mg (237 μmol)	1	1.27382	0.00087	0.3121	n.d.	3.121	n.d.	1.357	n.d.	(a-5)	(b-5)
		2	1.00172	0.00019	0.2287	n.d.	2.287	n.d.	0.994	n.d.	(a-6)	(b-6)
		3	1.27015	0.00258	0.2899	0.000189	2.899	0.001886	1.260	0.001	(a-6)	(b-6)

Supplementary Table 2 Absorption data for the quantification of NH_3 and N_2H_4 (2): Cluster **3** + HCl. Calibration curves are shown in Supplementary Figures 13-14.

	KC8	run	abs.		NH_3 (mM)		NH_3 (μmol)		equiv. per Ti-N=N-Ti		Calibration curve	
			NH_3	N_2H_4	NH_3	N_2H_4	NH_3	N_2H_4	NH_3	N_2H_4	NH_3	N_2H_4
[Ti-NN-Ti] 5 mg (2.3 μmol) +HCl	0 eq.	1	0.1839	0.08234	0.06995	0.006073	0.6995	0.06073	0.304	0.026	(a-7)	(b-7)
		2	0.1421	0.3689	0.05342	0.02713	0.5342	0.2713	0.232	0.118	(a-8)	(b-8)
		3	0.3352	0.7148	0.07486	0.05236	0.7486	0.5236	0.325	0.228	(a-9)	(b-9)
	2 mg (14.8 μmol)	1	0.2788	0.7029	0.1053	0.05078	1.053	0.5078	0.458	0.221	(a-3)	(b-3)
		2	0.3746	0.7175	0.1313	0.05208	1.313	0.5208	0.571	0.226	(a-1)	(b-1)
		3	0.2641	0.3951	0.06326	0.02836	0.6326	0.2836	0.275	0.123	(a-4)	(b-4)
	32 mg (237 μmol)	1	0.1089	0.00355	0.02668	0.000254	0.2668	0.00254	0.116	0.001	(a-5)	(b-5)
		2	0.2821	0.00018	0.06438	n.d.	0.6438	n.d.	0.28	n.d.	(a-6)	(b-6)
		3	0.2033	0.00067	0.0464	n.d.	0.464	n.d.	0.202	n.d.	(a-6)	(b-6)

$[NH_4]^+$ (mM)	abs.
0.1	0.25546
0.2	
0.3	0.87494
0.4	1.17769
0.5	1.39096

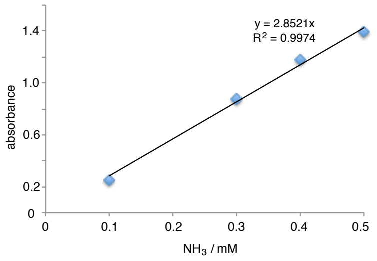

(a-1)

$[NH_4]^+$ (mM)	abs.
0.1	0.42295
0.2	0.81899
0.3	1.201
0.4	1.570
0.5	1.98159

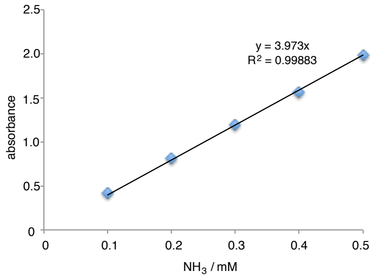

(a-2)

$[NH_4]^+$ (mM)	abs.
0.1	0.43223
0.2	0.88993
0.3	1.322
0.4	1.758
0.5	2.19306

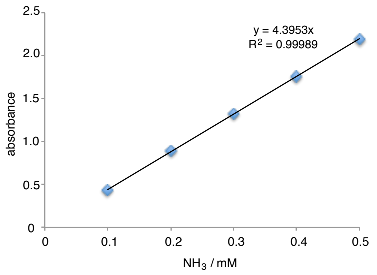

(a-3)

$[NH_4]^+$ (mM)	abs.
0.1	0.44076
0.2	0.84583
0.3	1.268
0.4	1.640
0.5	2.09321

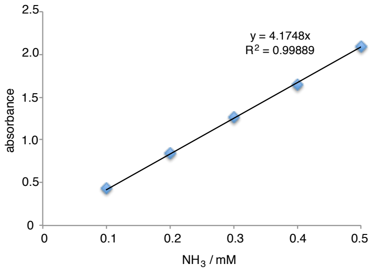

(a-4)

$[NH_4]^+$ (mM)	abs.
0.1	0.41855
0.2	0.84577
0.3	1.243
0.4	1.638
0.5	2.01183

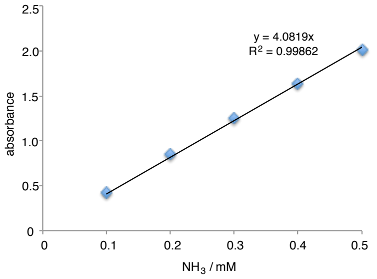

(a-5)

$[NH_4]^+$ (mM)	abs.
0.1	0.4317
0.2	0.85032
0.3	1.338
0.4	1.743
0.5	2.19548

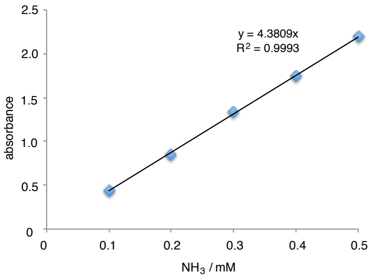

(a-6)

$[NH_4]^+$ (mM)	abs.
0.1	0.25133
0.2	0.53738
0.3	0.806
0.4	1.036
0.5	

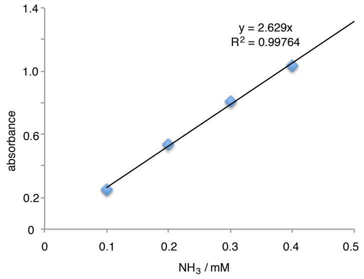

(a-7)

$[NH_4]^+$ (mM)	abs.
0.1	0.2619
0.2	0.53529
0.3	0.801
0.4	1.052
0.5	1.3377

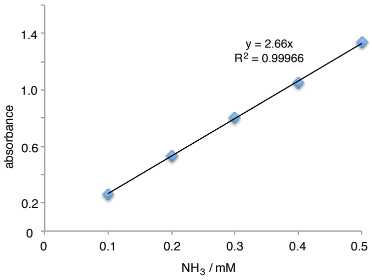

(a-8)

$[NH_4]^+$ (mM)	abs.
0.1	0.42387
0.2	0.94244
0.3	1.347
0.4	1.788
0.5	2.22512

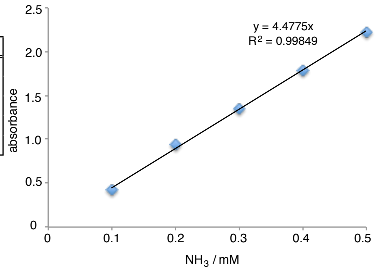

(a-9)

Supplementary Figure 13 Calibration curves for the quantification of NH_3. To maintain the accuracy of the analysis, the calibration curves were periodically recorded using fresh solutions for **A** and **B**.

$[N_2H_6]^{2+}$ (mM)	abs.
0.02	0.26212
0.04	0.54718
0.06	0.82228
0.08	1.11057
0.10	1.37769

(b-1)

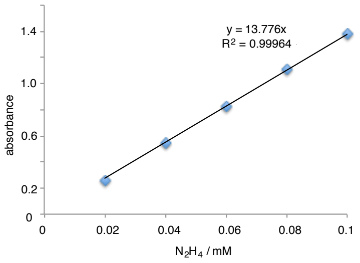

$[N_2H_6]^{2+}$ (mM)	abs.
0.02	0.26951
0.04	0.544
0.06	0.81652
0.08	1.098
0.10	1.37712

(b-2)

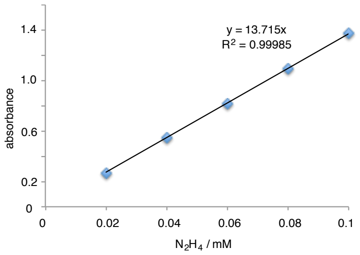

$[N_2H_6]^{2+}$ (mM)	abs.
0.02	0.26586
0.04	0.54773
0.06	0.82571
0.08	1.103
0.10	1.36837

(b-3)

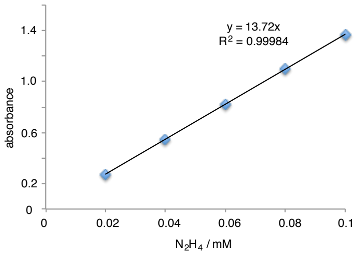

$[N_2H_6]^{2+}$ (mM)	abs.
0.02	0.28109
0.04	0.55889
0.06	0.83473
0.08	1.113
0.10	1.39359

(b-4)

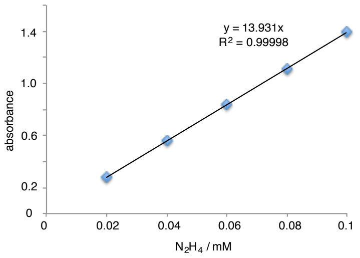

$[N_2H_6]^{2+}$ (mM)	abs.
0.02	0.28188
0.04	0.55963
0.06	0.84116
0.08	1.111
0.10	1.39673

(b-5)

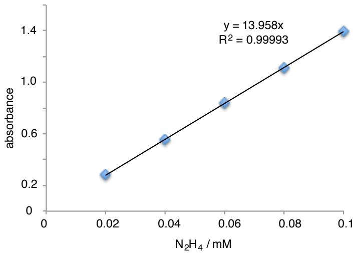

$[N_2H_6]^{2+}$ (mM)	abs.
0.02	0.27121
0.04	0.54488
0.06	0.82363
0.08	1.096
0.10	1.36742

(b-6)

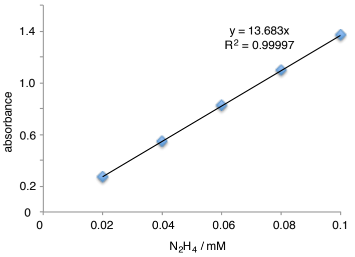

$[N_2H_6]^{2+}$ (mM)	abs.
0.02	0.25738
0.04	0.5357
0.06	0.81681
0.08	1.127
0.10	1.32567

(b-7)

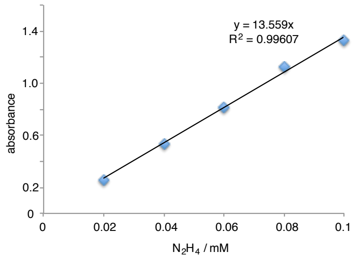

$[N_2H_6]^{2+}$ (mM)	abs.
0.02	0.26331
0.04	0.53534
0.06	0.81142
0.08	1.097
0.10	1.36031

(b-8)

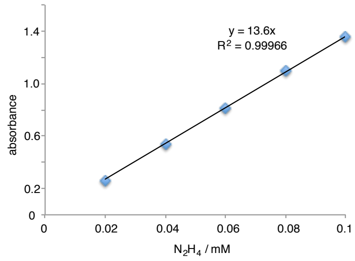

$[N_2H_6]^{2+}$ (mM)	abs.
0.02	0.2618
0.04	0.54213
0.06	0.81943
0.08	1.073
0.10	1.38389

(b-9)

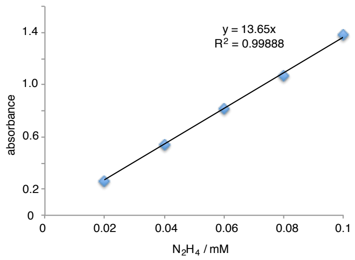

Supplementary Figure 14 Caribration curves for the quantification N_2H_4. To maintain the accuracy of the analysis, the calibration curves were periodically recorded using fresh solutions for **C** and **D**.

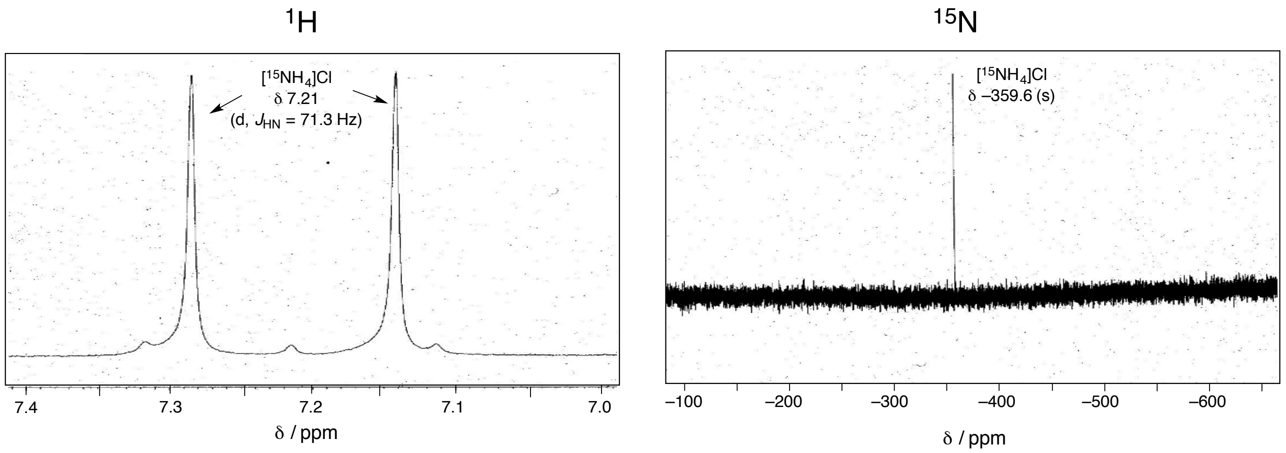

Supplementary Figure 15 The ^{1}H and $^{15}N\{^{1}H\}$ NMR spectra of $[^{15}NH_4][Cl]$ obtained from the protonation of ^{15}N-labeled **3** with H_2O in the presence of 100 equiv of KC_8 under an atmosphere of $^{14}N_2$.

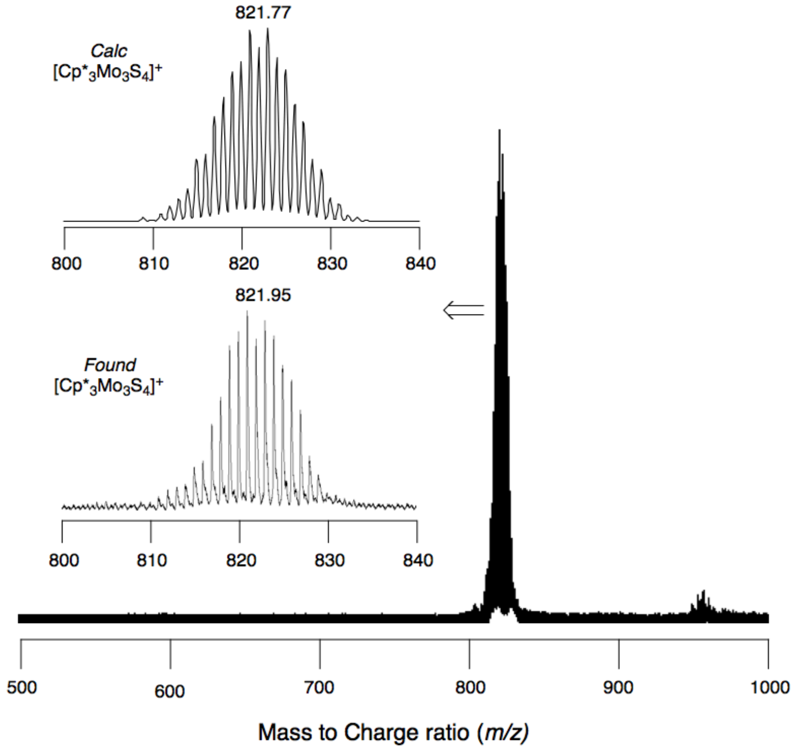

Supplementary Figure 16 ESI-MS spectrum of the THF solution obtained from the reaction of **3** with excess H_2O in the presence of 100 equiv of KC_8.

X-ray Crystal Structure Determination

The crystallographic data and refinement parameters for **2**, **3**, and [**3**]$^{-}$ are summarized in Supplementary Table 3. Single crystals were coated with oil (immersion Oil, type B: Code 1248, Cargille laboratories, Inc.) and mounted on loops. Diffraction data were collected at –100 ºC under a cold nitrogen stream on a Rigaku RA-Micro7 equipped with a Saturn70 CCD detector or a PILATUS 200K detector, using graphite-monochromated MoKα radiation (λ = 0.710690 Å). Six preliminary data frames were measured at 0.5° increments of ω in order to assess the crystal quality and preliminary unit cell parameters. The intensity images were also measured at 0.5° intervals of ω. The frame data were integrated using the CrystalClear program package, and the data sets were corrected for absorption using a REQAB program. The calculations were performed with the CrystalStructure program package. All structures were solved by direct methods, and refined by full-matrix least squares. The atomic coordinates for **2**, **3**, and [**3**]$^{-}$ have been deposited with the Cambridge Crystallographic Data Centre under reference nos. 1577330-1577332.

Supplementary Table 3. Crystallographic data for clusters **2**, **3**, and [**3**]$^{-}$.

	**2**·CH_2Cl_2	**3**	[**3**]$^{-}$
Formula	$C_{31}H_{47}Cl_4Mo_3S_4Ti$	$C_{84}H_{138}N_2O_6K_2Mo_6S_8Ti_2$	$C_{84}H_{138}N_2O_6K_3Mo_6S_8Ti_2$
Fw (g mol^{-1})	1025.44	2278.08	2317.18
Crystal system	Triclinic	Orthorhombic	Orthorhombic
Space group	*P*-1 (#2)	*Pnma* (#62)	*Pnma* (#62)
a (Å)	10.3456(10)	23.602(2)	23.2696(17)
b (Å)	11.2061(12)	20.672(2)	20.8094(14)
c (Å)	16.7227(16)	19.846(2)	20.3010(13)
α (deg)	86.845(5)	90	90
β (deg)	84.230(5)	90	90
γ (deg)	85.090(4)	90	90
V (Å^3)	1919.7(3)	9682.9(16)	9830.3(12)
Z	2	4	4
D_{calc} (g cm^{-3})	1.774	1.563	1.509
μ (Mo Kα) (cm^{-1})	1.676	1.213	1.232
$2\theta_{max}$ (deg)	55.0	55.0	55.0
No. of measured reflections	Total: 32338	Total: 74489	Total: 154332
No. of Observation	8732	11362	11634
No. of Variables	462	566	703
$R1^a$	0.0212	0.0487	0.0371
$wR2^b$	0.0530	0.1493	0.1150
GOF^c	1.043	0.962	1.067

$^aR_1 = \Sigma|F_o|-|F_c|/\Sigma|F_o|$ ($I > 2\sigma(I)$). $^bwR_2 = [(\Sigma(w(|F_o|-|F_c|)^2/\Sigma wF_o^2))^{1/2}$ (all reflections).

^{c}GOF $= [\Sigma w(|F_o|-|F_c|)^2/(N_o-N_v)]^{1/2}$ (where N_o = number of observations, N_v = number of variables).
